# Tumour volume is a predictor of lymphovascular invasion in differentiated small thyroid cancer

**DOI:** 10.1530/EO-22-0066

**Published:** 2022-09-22

**Authors:** Krishna Vikneson, Tariq Haniff, May Thwin, Ahmad Aniss, Alex Papachristos, Mark Sywak, Anthony Glover

**Affiliations:** 1 Department of Endocrine Surgery, Royal North Shore Hospital, Northern Sydney Local Health District, Sydney, New South Wales, Australia; 2 The Kinghorn Cancer Centre, Garvan Institute of Medical Research, St. Vincent’s Clinical School, Faculty of Medicine, University of New South Wales Sydney, Sydney, New South Wales, Australia; 3 Sydney Medical School, Faculty of Medicine and Health, University of Sydney, Sydney, New South Wales, Australia

**Keywords:** differentiated thyroid cancer, endocrine surgery, surgical outcomes, risk stratification, tumour volume

## Abstract

**Objectives:**

For small thyroid cancers (≤2 cm), tumour volume may better predict aggressive disease, defined by lymphovascular invasion (LVI) than a traditional single measurement of diameter. We aimed to investigate the relationship between tumour diameter, volume and associated LVI.

**Methods:**

Differentiated thyroid cancers (DTC) ≤ 2 cm surgically resected between 2007 and 2016 were analysed. Volume was calculated using the formula for an ellipsoid shape from pathological dimensions. A ‘larger volume’ cut-off was established by receiver operating characteristic (ROC) analysis using the presence of lateral cervical lymph node metastasis (N1b). Logistic regression was performed to compare the ‘larger volume’ cut-off to traditional measurements of diameter in the prediction.

**Results:**

During the study period, 2405 DTCs were surgically treated and 523 met the inclusion criteria. The variance of tumour volume relative to diameter increased exponentially with increasing tumour size; the interquartile ranges for the volumes of 10, 15 and 20 mm diameter tumours were 126, 491 and 1225 mm^3,^ respectively. ROC analysis using volume to predict N1b disease established an optimal volume cut-off of 350 mm^3^ (area under curve = 0.59, *P* = 0.02) as ‘larger volume’. ’Larger volume’ DTC was an independent predictor for LVI in multivariate analysis (odds ratio (OR) = 1.7, *P* = 0.02), whereas tumour diameter > 1 cm was not (OR = 1.5, *P* = 0.13). Both the volume > 350 mm^3^ and dimension > 1 cm were associated with greater than five lymph node metastasis and extrathyroidal extension.

**Conclusion:**

In this study for small DTCs ≤ 2 cm, the volume of >350 mm^3^ was a better predictor of LVI than greatest dimension > 1 cm.

## Introduction

Well-differentiated thyroid cancer (DTC) and papillary thyroid cancer (PTC) incidence has been increasing in comparison to other thyroid cancers (Davies & Welch 2014, Davies & Hoang 2021). However, most thyroid cancer diagnoses are unlikely to be life-threatening with a greater than 95% 10-year overall survival (Ito *et al.* 2018, Park *et al.* 2018). Most of the rise in new cases seem to be small and lower risk cancers (Guth *et al.* 2009, Dal Maso *et al.* 2018). Management of these cancers is controversial, with treatment recommendations ranging from active surveillance (AS) to surgery and the use of radioactive iodine therapy (Boucai *et al.* 2017, Zanocco *et al.* 2019). While for some patients more aggressive treatment can be indicated or preferred, it has been argued that with conservative approaches the same or better overall outcomes may be achieved with careful patient selection (Tuttle 2018).

Pre-operative assessment and prediction of high-risk cancers is important when counselling patients to facilitate shared decision-making in deciding on a treatment approach. Currently, the major risk stratification and staging systems used are the 2015 American Thyroid Association (ATA) risk scoring system (Haugen *et al.* 2016) and the 8th American Joint Committee on Cancer (AJCC) staging systems (Edge 2017), and both use the greatest diameter of the tumour to quantify size. However, DTCs have been reported to be ellipsoid in shape rather than spherical, so tumour volume may be a better representation of a tumour’s size (Pennington *et al.* 2018). Volume may be a better predictor of the tumour’s pathological characteristics and hence may allow better pre-operative risk stratification. Currently, the ATA guidelines (Haugen *et al.* 2016) combine clinical and pathological features to estimate recurrence risk.

Pathological findings such as the presence of lymphovascular invasion (LVI) and lymph node metastases often upstage the patient to an intermediate risk with the presence of LVI thought to increase the risk of recurrence by 15–30% (Haugen *et al.* 2016); hence, completion thyroidectomy is often necessary after review of the initial post-operative histology. While the presence of nodal metastases is a factor that may be able to be determined pre-operatively, LVI is not. So if we are able to better predict for LVI, it may allow for better risk assessment from the time of diagnosis.

Prior research examining DTCs of all sizes found that volume correlates strongly with nodal metastasis, better than the greatest diameter alone (Pennington *et al.* 2018). Similarly, in AS studies of DTC, it has been established that serial volume measurements can allow the prediction of growth characteristics earlier than measurements of the greatest diameter (Tuttle *et al.* 2017). Tumour volume studies have also been shown to be an independent predictor of outcomes in other cancers such as early stage rectal cancer (Jiang *et al.* 2018), non-small cell lung cancer (Takenaka *et al.* 2016, Su *et al.* 2017) and prostate cancer (Stamey *et al.* 1999).

This study aimed to evaluate the relationship of pathological tumour volume to greatest dimension for small (≤2 cm) DTC and assess if volume correlates with post-operative risk factors for recurrence such as LVI.

## Methodology

### Study design

The study aimed to evaluate whether risk stratification of small DTCs was possible based on tumour volume as opposed to greatest diameter. A retrospective study analysis of consecutive, surgically treated DTC patients at Royal North Shore Hospital was performed after approval was obtained from the local human research ethics committee (Northern Sydney Local Health District). Data were extracted from the prospectively maintained endocrine surgery database and patients were included if they underwent surgery and had pathologically confirmed DTC with a diameter of ≤20 mm. For patients with multifocal cancers, the largest tumour by greatest dimension was used. Patients were excluded from the analysis if they did not have all three dimensions of their tumour histologically reported or were classified as high risk as per the 2015 ATA risk of recurrence guidelines (Haugen *et al.* 2016).

### Operative treatment

All patients included in this study underwent surgery within a tertiary unit with a pathological assessment performed by pathologists with endocrine cancer expertise. Patients also had both known and incidental thyroid cancers, with select patients undergoing central lymph node dissection for either prophylactic or therapeutic intent and if indicated lateral lymph node dissection for therapeutic intent.

### Calculating tumour volume

All pathological assessment of tumours was reported independently by our pathologists. Tumour volume was calculated assuming an ellipsoid shape using the diameter method (volume = length (mm) × width (mm) × height (mm) × π/6) as that is the clinical impression of the shape and method used in previous endocrine surgery studies (Tuttle *et al.* 2017, Pennington *et al.* 2018).

### Outcome measures

The primary endpoint was the presence of LVI, While other risk factors analysed included pathologically confirmed presence of more than five metastatic lymph nodes, presence of lateral cervical nodal metastasis (N1b) and minor extrathyroidal extension (ETE). Minor ETE was defined as spreading that extends focally out of the thyroid into the adjacent soft tissue but not widespread into the soft tissue or skeletal muscle.

### Establishing the optimal cut-off

To form a hypothetical volume cut-off, a receiver operating characteristic (ROC) curve analysis was performed for different outcomes including N1b. The optimal cut-off point was determined to be the point closest to the top left corner indicating the optimal combination of sensitivity and specificity, as described in other studies (Unal 2017). A separate subgroup excluding patients with N1b disease was conducted using the initial cut-off to see if volume had the same utility.

### Statistical analysis

Statistical tests were conducted using IBM SPSS Statistics Version 27.0 (IBM). To evaluate the associations between clinicopathological characteristics and tumour size, Fisher’s exact test or chi-squared test was used for categorical variables, while the association with age (the only continuous variable) was assessed with the Student’s *t*-test. Binary logistic regression was conducted at selected endpoints. A histogram was created to illustrate the tumour volume distribution of the cohort. Both linear and exponential regression analyses were performed to explore the relationship between tumour volume and greatest dimension. For all statistical analyses, a two-sided *P*-value < 0.05 was considered to be statistically significant.

## Results

### Patient demographics

Between March 2007 and December 2016, a total of 2405 patients underwent surgery for DTC. Patients who had tumours with the greatest dimension > 20 mm (*n*  = 708), those who did not have all three dimensions pathologically reported (*n*  = 1164), or those who were classified as high-risk as per the 2015 ATA risk of recurrence (*n*  = 10) were excluded. A total of 523 patients were identified for final analysis (Supplementary Fig. 1, see section on supplementary materials given at the end of this article) with included patients’ demographics summarised in Table 1.

**Table 1:** Baseline patient demographics and clinicopathological characteristics.

Characteristics	Number of patients
Age (years) mean age 50.0, range 13–85	
≤55	195 (37.3%)
>55	328 (62.7%)
Sex	
Male	101 (19.3%)
Female	422 (80.7%)
Hashimoto’s thyroiditis	
Yes	38 (7.3%)
No	485 (92.7%)
Type of operation	
Thyroidectomy	500 (95.6%)
Hemithyroidectomy	23 (4.4%)
Histological type	
Papillary cancer	488 (93.3%)
Follicular cancer	24 (4.6%)
Hurthle cell cancer	11 (2.1%)
Incidental	
Yes	431 (82.4%)
No	92 (17.6%)
Lymphovascular invasion	
No	385 (73.6%)
Yes	138 (26.4%)
Total positive lymph nodes	
≤5	464 (88.7%)
>5	59 (11.3%)
Lateral lymph node disease (N1b)	
No	454 (86.8%)
Yes	69 (13.2%)
Minor extrathyroidal extension	
No	418 (79.9%)
Yes	105 (20.1%)

The mean age of the cohort was 50 years (range: 13–85), with 422 (80.7%) patients being female. Total thyroidectomy was performed on 500 patients (96%) while the remaining 23 (4%) had a hemithyroidectomy. A total of 397 (75.9%) patients had central lymph node dissection and 181 (34.6%) had lateral lymph node dissection. There were 431 patients (82.4%) who had pre-operatively known DTC and the remaining 92 (17.6%) had incidental DTC found after operation for other indications (Table 1). The major histological type diagnosed was papillary cancer (93%) followed by follicular thyroid cancer (5%) and Hurthle cell carcinoma (2%).

A total of 170 patients (32.5%) had a micro-DTC (≤10 mm) and 353 (67.5%) with greatest dimension between 10 and 20 mm. Both patients treated for cancers (≤10 mm) and 10–20 mm were similar except for a greater number of patients treated with total thyroidectomy with larger cancers (Supplementary Table 1).

### Relationship of greatest diameter to tumour volume

**Figure 1:**
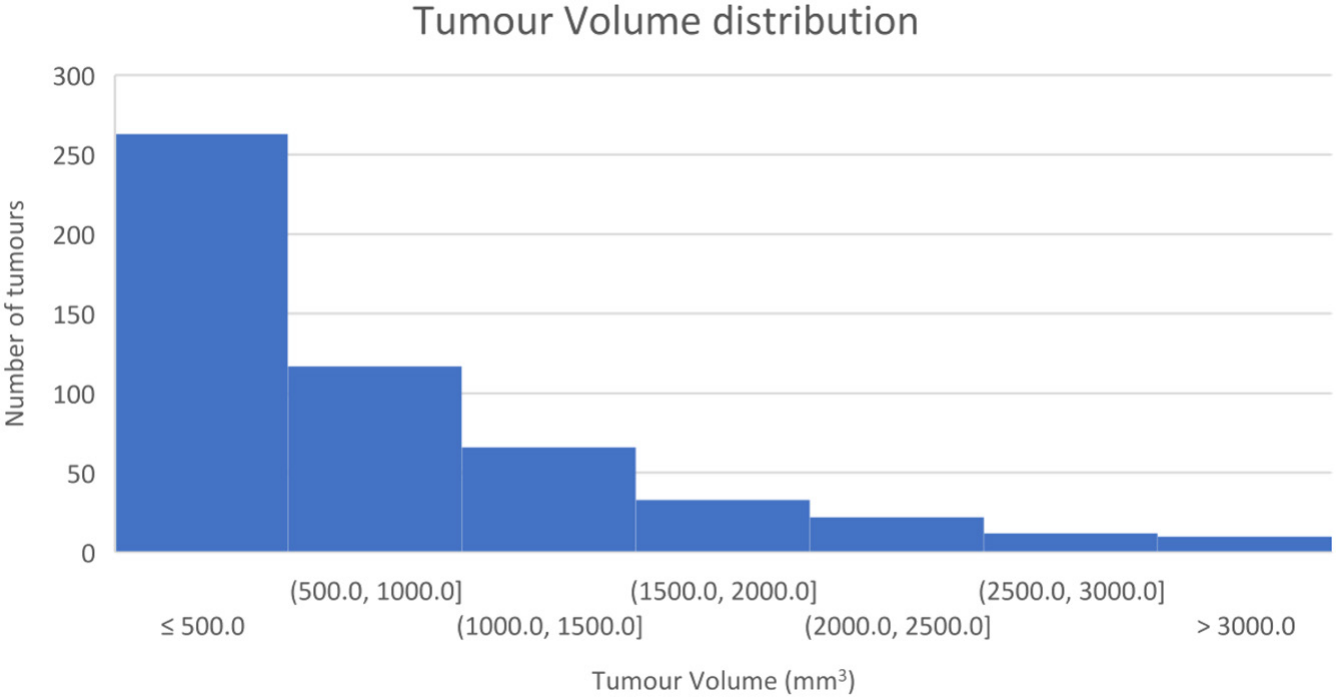
Histogram of the tumour volume distribution for the cohort.

**Figure 2:**
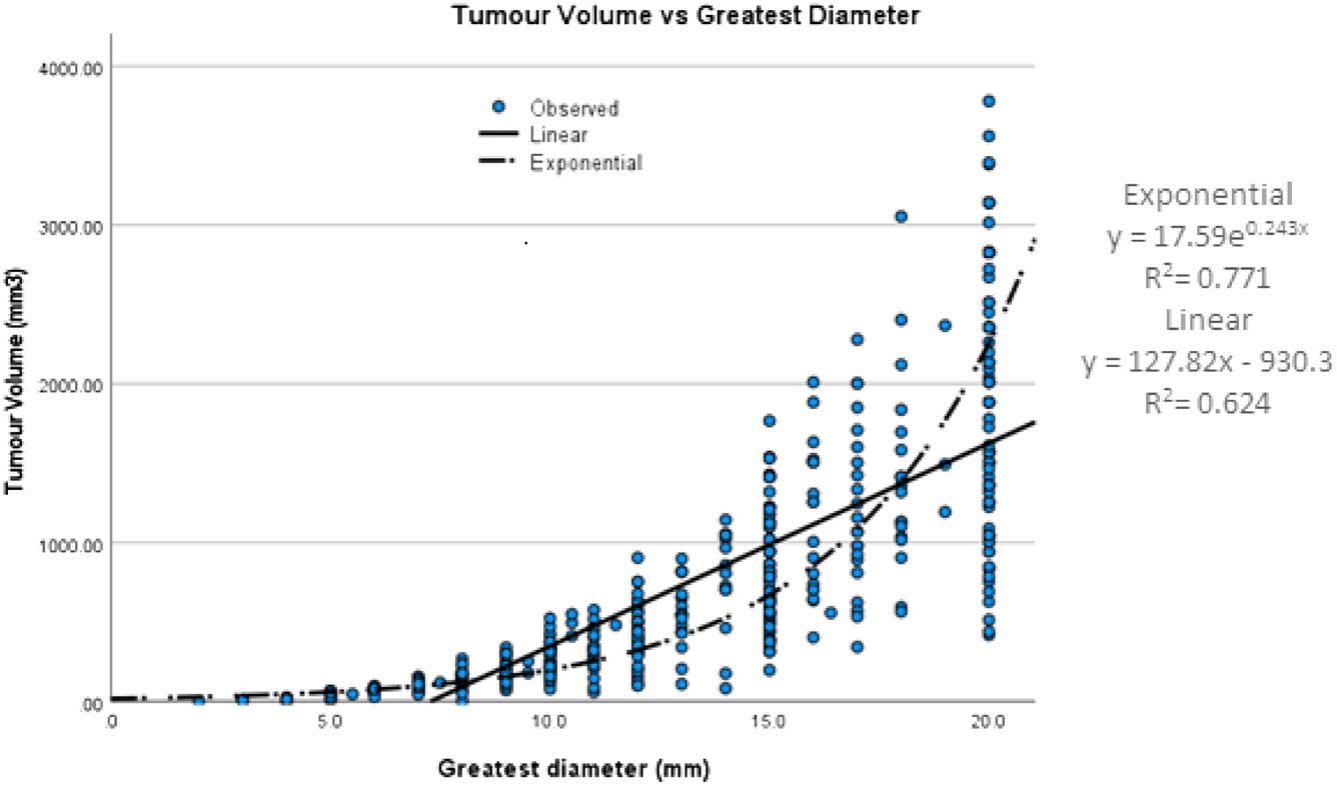
Regression analysis of greatest diameter and tumour volume.

Tumour volume ranged from 0.3 to 3780.4 mm^3^, with a median of 494.8 mm^3^ (with Q1–Q3 209.4–1047.2 mm^3^). The distribution of volumes was positively skewed as shown in the histogram plot (Fig. 1). Regression analysis between tumour volume and greatest dimension, and the correlation analysis of both exponential and linear model fits are presented in Fig. 2. The results suggest that the correlation between tumour volume and greatest dimension is better explained using an exponential growth model compared to linear model (r^2^ = 0.771 vs 0.624).

**Figure 3:**
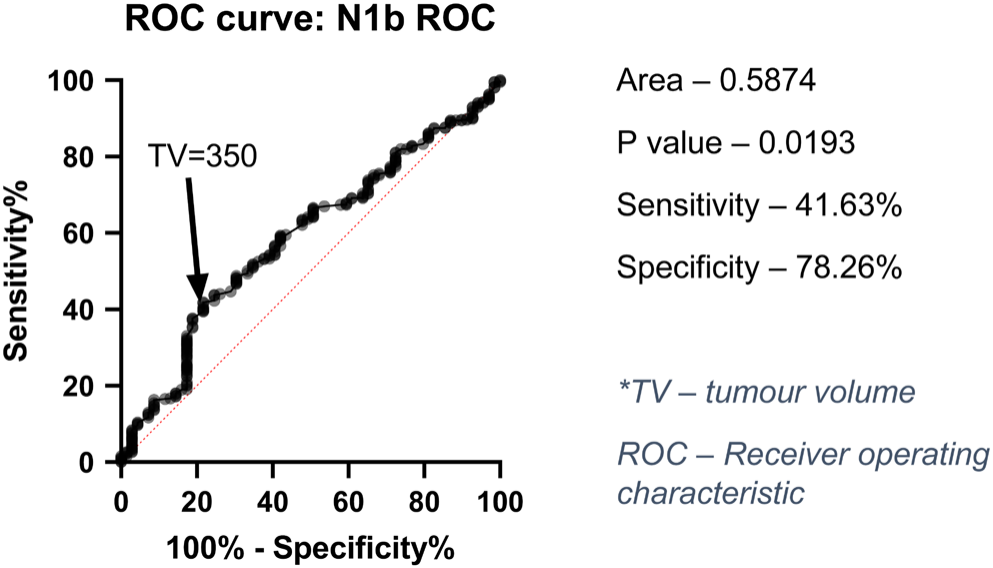
Receiver operating characteristic curve for tumour volume as a predictor of lateral lymph node disease (N1b).

As the greatest dimension increases, the variance between tumour volume and tumour diameter increased, with inter-quartile ranges (IQR) for volume measurements of 126, 491 and 1225 mm^3^ for tumour diameters of 10, 15 and 20 mm, respectively. The ROC curve (Fig. 3) analysis for predicting N1b established the optimal tumour volume cut-off value to be 350 mm^3^ (sensitivity= 41.6%, specificity= 78.26%) with the area under the curve being 0.587 (*P*  = 0.019). Using the exponential equation from Fig. 2, a tumour volume of 350 mm^3^ corresponds to a maximum diameter measurement of 12.3 mm.

### Recategorizing tumours and association of LVI with small and large volume groups

The 350 mm^3^ cut-off was used to classify two volume groups – a larger volume group of tumours > 350 mm^3^ and a smaller volume group of tumours ≤ 350 mm^3^. A total of 39 (11%) tumours that were >10 mm in greatest diameter were recategorized into the smaller volume group, while 10 (6%) micro-DTC tumours were recategorized into the larger volume group. This resulted in 199 patients (38.0%) in the small tumour volume group and 324 patients (62.0%) in the large tumour volume group.

Assessing the various tumour risk factors and clinicopathological characteristics, both volume and greatest diameter had statistical differences between sizes for LVI and the presence of five or more positive lymph nodes (Table 2). The greatest diameter cut-off (>10 mm) was not able to predict for presence of N1b lymph node disease, whereas tumour volume group did (*P* =  0.003 vs 0.097). Conversely, greatest diameter (≤10 mm) could predict for minor extrathyroidal extension (*P* =  0.036 vs 0.056).

**Table 2:** Association between clinicopathological characteristics using micro-DTC cut-off and tumour volume groups created.

Clinicopathological characteristic	Using micro-DTC cut-off			Using tumour volume groups created		
	Greatest diameter		*P* value	Small tumour volume group (*n* = 199)	Large tumour volume group (*n* = 324)	*P* value
	≤10 mm (*n* = 170)	>10 mm (*n* = 353)				
Lymphovascular invasion						
No	136 (80.0%)	249 (70.5%)	**0.026**	163 (81.9%)	222 (68.5%)	**0.007**
Yes	34 (20.0%)	104 (29.5%)		36 (18.1%)	102 (31.5%)	
Total positive lymph nodes						
≤5	163 (95.9%)	301 (85.3%)	<0.001	190 (95.5%)	274 (84.6%)	<0.001
>5	7 (4.1%)	52 (14.7%)		9 (4.5%)	50 (15.4%)	
Lateral lymph node disease (N1b)						
No	154 (90.6%)	300 (85.0%)	**0.097**	184 (92.5%)	270 (83.3%)	**0.003**
Yes	16 (9.4%)	53 (15.0%)		15 (7.5%)	54 (16.7%)	
Minor extrathyroidal extension						
No	145 (85.3%)	273 (77.3%)	**0.036**	168 (84.4%)	250 (77.2%)	**0.056**
Yes	25 (14.7%)	80 (22.7%)		31 (15.6%)	74 (22.8%)	

micro-DTC, micro-differentiated thyroid cancers.

The univariate and multivariate analyses for predicting the presence of LVI are shown in Table 3 (A) and (B) using volume and greatest diameter, respectively. In the univariate analysis, the presence of more than five metastatic lymph nodes (OR = 5.555, *P* = <0.001) and minor ETE (OR = 2.358, *P* = <0.001) were both associated with LVI. Both greatest diameter > 10 mm (OR = 1.671, *P* = 0.022) and volume > 350 mm^3^ (OR = 2.080, *P* = 0.001) were significant in the univariate analysis. However, in the multivariate analysis, only volume > 350 mm^3^ remained significant as an independent predictor of LVI (OR = 1.7, *P* = 0.02).

**Table 3:** Univariate and multivariate regression analysis for prediction of LVI.

Variable	Univariate analysis		Multivariate analysis	
	Odds ratio (95% CI)	*P*-value	Odds ratio (95% CI)	*P*-value
(A) Lymphovascular invasion and tumour volume				
Female sex	0.814 (0.504–1.315)	0.400		
Age > 55	0.725 (0.480–1.096)	0.127		
Hashimoto’s disease	0.996 (0.471–2.108)	0.992		
>5 Involved lymph nodes	5.555 (3.150–9.798)	<0.001	4.563 (2.546–8.178)	<0.001
Minor extrathyroidal extension	2.358 (1.502–3.703)	<0.001	1.993 (1.237–3.210)	0.005
Tumour volume > 350 mm^3^	2.080 (1.353–3.199)	0.001	1.678 (1.072–2.627)	0.024
Multifocality	1.112 (0.753–1.641)	0.595		
(B) Lymphovascular invasion and greatest diameter				
Female sex	0.814 (0.504–1.315)	0.400		
Age > 55	0.725 (0.480–1.096)	0.127		
Hashimoto’s disease	0.996 (0.471–2.108)	0.992		
>5 Involved lymph nodes	5.555 (3.150–9.798)	<0.001	4.807 (2.685–8.607)	0.004
Minor extrathyroidal extension	2.358 (1.502–3.703)	<0.001	2.017 (1.254–3.244)	<0.001
Greatest diameter > 10 mm	1.671 (1.076–2.594)	0.022	1.314 (0.830–2.080)	0.244
Multifocality	1.112 (0.753–1.641)	0.595		

### Analysis of patients without lateral nodal disease

By using the volume cut-off of 350 mm^3^, a separate analysis was conducted on the same cohort after excluding all N1b patients (*n*  = 69) which left a cohort of 454 patients.

The multivariate analysis for LVI confirmed that the volume cut-off of 350 mm^3^ remained a significant predictor (OR = 1.840, *P*  =  0.029) while the larger greatest diameter (*P* =  0.133) group was not significant (Table 4A andB).

**Table 4:** Univariate and multivariate regression analyses for prediction of LVI excluding N1b patients.

Variable	Univariate analysis		Multivariate analysis	
	Odds ratio (95% CI)	*P*-value	Odds ratio (95% CI)	*P*-value
(A) Lymphovascular invasion and tumour volume				
Female sex	1.043 (0.572–1.905)	0.890		
Age > 55	0.845 (0.521–1.361)	0.489		
Hashimoto’s disease	0.414 (0.123–1.393)	0.154		
>5 Involved lymph nodes	2.634 (1.057–6.563)	0.038	2.312 (0.912–5.860)	0.077
Minor extrathyroidal extension	2.161 (1.255–3.720)	0.005	2.095 (1.209–3.631)	0.008
Tumour volume > 350 mm^3^	1.944 (1.132–3.341)	0.016	1.840 (1.064–3.183)	0.029
Multifocality	1.103 (0.695–1.752)	0.676		
(B) Lymphovascular invasion and greatest diameter				
Female sex	1.043 (0.572–1.905)	0.890		
Age > 55	0.845 (0.521–1.361)	0.489		
Hashimoto’s disease	0.414 (0.123–1.393)	0.154		
>5 Involved lymph nodes	2.634 (1.057–6.563)	0.038	2.277 (0.894–5.802)	0.085
Minor extrathyroidal extension	2.161 (1.255–3.720)	0.005	2.094 (1.210–3.623)	0.008
Tumour diameter > 10 mm	1.638 (1.974–2.754)	0.063	1.501 (0.884–2.550)	0.133
Multifocality	1.103 (0.695–1.752)	0.676		

## Discussion

With smaller DTCs having a generally favourable prognosis, treatment has been trending towards more passive approaches such as AS and hemithyroidectomy (Haugen *et al.* 2016, Russ *et al.* 2017) to minimise surgical morbidity. This study looked at whether tumour volume could be a better indicator for determining if a patient with a small DTC is a candidate for less aggressive treatment. While macroscopic lymph node involvement can be assessed using ultrasound pre-operatively, LVI is only able to be determined post-operatively on histopathological examination (DiMarco *et al.* 2019). Thus, utilising a factor that may predict LVI as an endpoint may be more useful for pre-operative work-up of a patient.

This study examined whether tumour volume is a better predictor of LVI than traditional size measurements. The study cohort was 81% female which is in line with the typical approximate 4:1 female to male ratio reported in other endocrine surgery studies (Sanders & Cady 1998, LeClair *et al.* 2021). The 93.3% proportion of PTCs in this cohort is comparable to the literature (Yip & Sosa 2016, Grani *et al.* 2018).

This study shows that as the greatest tumour dimension increases, there is increasing variation in tumour volume. A study by Pennington *et al.* used sonographic volumetric assessment and found similar findings for their larger tumours – true tumour size or volume was being misrepresented by taking a unidimensional greatest dimension measurement (Pennington *et al.* 2018). While Pennington *et al.* used pre-operative sonography data and this study used pathological data, pre-operative thyroid sonography has been shown to correlate well and have a very high positive predictive value agreement with post-operative histopathological assessment (Park *et al.* 2009, Moon *et al.* 2011) in terms of greatest diameter.

Our regression analysis showed that the relationship between volume and greatest dimension was better explained by an exponential rather than a linear model. Our results show there were 10 mm greatest dimension tumours that had a greater volume than many than 11, 12 and even some 20 mm greatest dimension tumours (Fig. 2). This could explain previous research that tumour staging with greatest diameter is not correlated precisely with true tumour burden (Ball *et al.* 2006).

ROC curve analysis showed a tumour volume of 350 mm^3^ was the optimal cut-off in predicting N1b disease. By creating a cut-off, all ≤20 mm greatest diameter tumours were able to be separated into two volume groups, and this was compared with the current DTC T1a and T1b staging which uses the greatest diameter = 10 mm cut-off by AJCC (Edge 2017). The study by Park *et al.* on tumour volume as a predictor for occult central lymph node metastasis in PTC also performed a ROC analysis to determine the optimal tumour volume cut-off (Park *et al.* 2015). This was found to be 385 mm^3^ which is close to what was established in our cut-off.

The tumour volume cut-off point of 350 mm^3^ corresponds to greatest diameter of 12.3 mm which is slightly greater than the current cut-off for micro-DTC of 10 mm. To our knowledge, there have not been any studies that investigated the relationship between volume and LVI. Tumour volume has shown promise in AS studies as an early indicator for cancer growth kinetics (Tuttle *et al.* 2017). Furthermore, Lim *et al.* (2017) found that pre-operative tumour volume measurements can be more useful than greatest diameter for predicting the risk of recurrence. However, the aforementioned studies excluded patients with higher risk pathological features such as LVI or ETE (Lim *et al.* 2017, Tuttle *et al.* 2017).

Our rate of LVI was 26.4% which lies between previous studies’ incidence rates from different institutes, with a varying incidence of LVI of 3–47% (Nishida *et al.* 2002, Falvo *et al.* 2005, Mete & Asa 2011, Wreesmann *et al.* 2015). Our results indicated that a pathological tumour volume of >350 mm^3^ was an independent predictor of LVI on multivariate analysis. The other predictive factors for LVI were greater than five metastatic lymph nodes and minor ETE, which concurs with previous studies and suggests that LVI typically occurs in combination with other clinicopathological indicators (Wreesmann *et al.* 2015). Interestingly, the age cut-off did not have a statistically significant correlation with LVI, in contrast to previous studies (Can *et al.* 2015, Sezer *et al.* 2017).

Further to these findings, only tumour volume was a significant predictor for LVI after excluding N1b patients. This is significant as N1b was used to create the optimal cut-off for tumour volume; hence showing the cut-off still holds without this subset of patients suggests it still holds utility. In addition, N1b patients may be able to be identified pre-operatively and, posing a greater risk, would not be suitable for a passive treatment approach.

There are some limitations to this study; since all three dimensions are not routinely reported in final histopathological analysis, there may be some selection bias. This project also optimised the volume cut-off in terms of N1b which may also be a source of bias. However, this project was intended as a pilot study to support further research to investigate the relationship between pre-operative radiological volume measurements with pathological risk stratification. Finally, we used the outcome of LVI as a surrogate endpoint, and future research should investigate the relationship of volume to clinical outcomes such as recurrence.

## Conclusion

This study showed that there is a wide range of tumour volumes associated with a single measure of greatest dimension and that tumour volume is a better predictor of LVI. The results suggest that volume has the potential to be used as an indicator for factors that cannot yet be reliably predicted or determined pre-operatively such as LVI. Since volume can be obtained pre-operatively by radiological assessment, routine volume measurements may allow clinicians to perform a more accurate preoperative risk assessment and facilitate clinical management decision.

## Supplementary Material

Supplementary Material

## Declaration of interest

The authors declare that there is no conflict of interest that could be perceived as prejudicing the impartiality of the research reported.

## Funding

Anthony Glover is supported by a Cancer Institute NSWhttp://dx.doi.org/10.13039/501100001171 Early Career Fellowship (2019/ECF1081) for this project.
