# Supplementary material for: Tumour volume is a predictor of lymphovascular invasion in differentiated small thyroid cancer
Source: Endocr Oncol. 2022 Sep 22;2(1):42–9. doi: 10.1530/EO-22-0066 (PMC10259346; doi:10.1530/EO-22-0066)
Supplement: Supplementary Material [file supplementary_material.pdf]

Supplementary material

Supplementary table 1: Association between population characteristics and microDTC cut-off

Variable	Patients with Greatest Diameter		P value
	**≤10mm (n= 170)**	**>10mm (n=353)**	
**Age**	Mean 49.1, Range 14-84	Mean 50.5, Range 13-85	0.947
≤55	58 (34.1%)	138 (39.1%)	0.290
>55	112 (65.9%)	215 (60.9%)	
**Sex**			0.723
Male	31 (18.2%)	70 (19.8%)	
Female	139 (81.8%)	283 (80.2%)	
**Hashimoto's Thyroiditis**			0.107
Yes	17 (10.0%)	21 (5.9%)	
No	153 (90.0%)	332 (94.1%)	
**Type of operation**			<0.001
Thyroidectomy	153 (90.0%)	347 (98.3%)	
Hemithyroidectomy	17 (10.0%)	6 (1.7%)	
**Histological type**			0.702
Papillary Cancer	160 (94.1%)	328 (92.9%)	
Follicular Cancer	6 (3.5%)	18 (5.1%)	
Hurthle Cell Cancer	4 (2.4%)	7 (2.0%)	

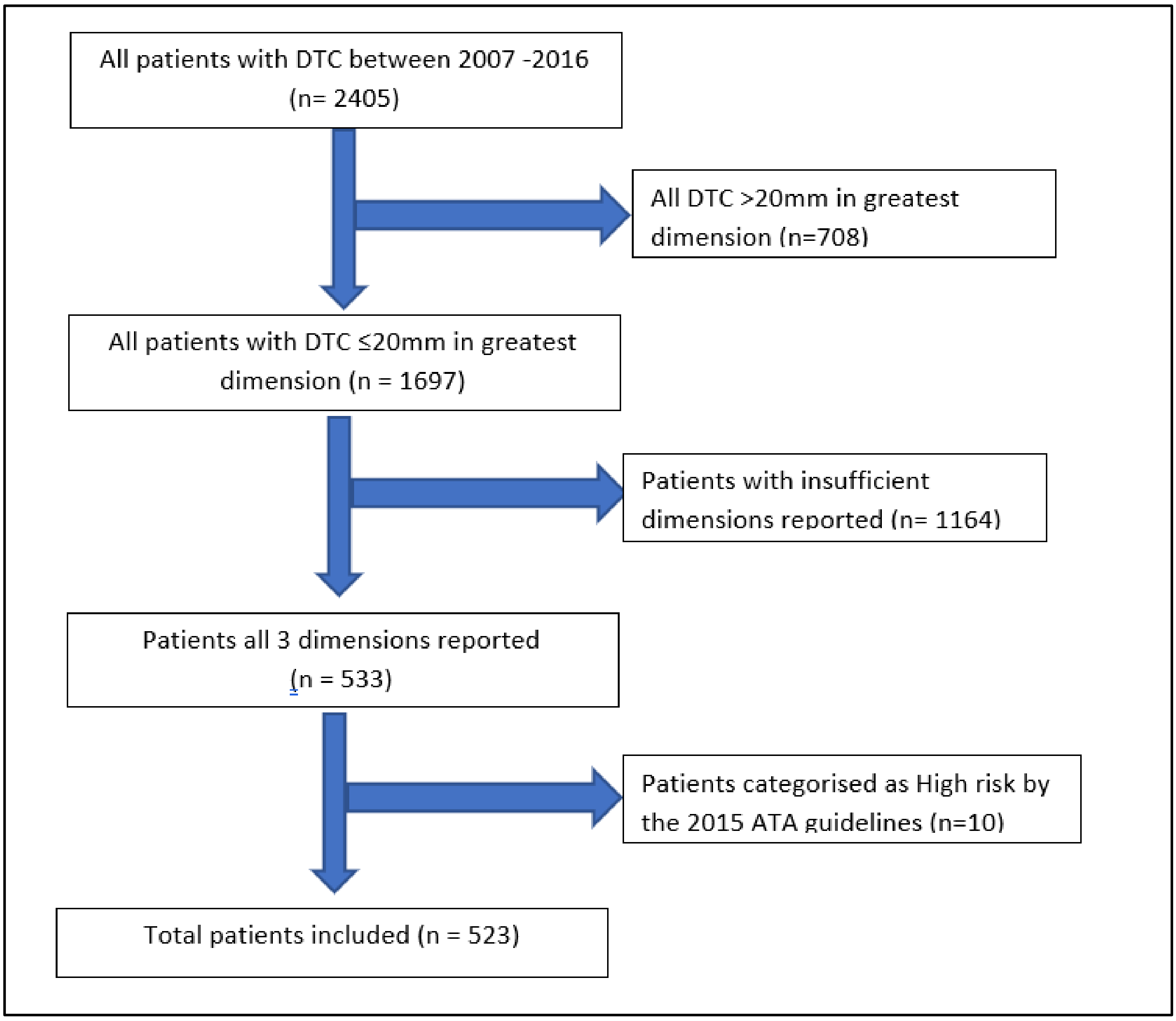

Supplementary Figure S1: Flowchart of inclusion and exclusion criteria of the study sample.

**ATA – American Thyroid Association*

***DTC – Differentiated thyroid cancer*
